# Low BRMS1 expression promotes nasopharyngeal carcinoma metastasis in vitro and in vivo and is associated with poor patient survival

**DOI:** 10.1186/1471-2407-12-376

**Published:** 2012-08-29

**Authors:** Qing-Mei He, Wen-Fei Li, Bi-Jun Huang, Ying Sun, Ling-Long Tang, Mo Chen, Ning Jiang, Lei Chen, Jing-Ping Yun, Jing Zeng, Ying Guo, Hui-Yun Wang, Jun Ma

**Affiliations:** 1 Department of Radiation Oncology, Sun Yat-sen University Cancer Center, Guangzhou, People’s Republic of China; 2 Present address: State Key Laboratory of Oncology in South China, Sun Yat-sen University Cancer Center, 651 Dongfeng Road East, Guangzhou 510060, Peoples Republic of China; 3 Department of Pathology, Sun Yat-sen University Cancer Center, Guangzhou, Peoples Republic of China; 4 Department of National Clinical Study Center for Anticancer Drugs, Sun Yat-sen University Cancer Center, Guangzhou, Peoples Republic of China; 5 Department of Radiation Oncology State Key Laboratory of Oncology in South China, Sun Yat-sen University Cancer Center, 651 Dongfeng Road East, Guangzhou, 510060, Peoples Republic of China

**Keywords:** BRMS1, Nasopharyngeal carcinoma, Metastasis, Prognosis

## Abstract

**Background:**

Breast cancer metastasis suppressor 1 (BRMS1) is a metastasis suppressor gene. This study aimed to investigate the impact of BRMS1 on metastasis in nasopharyngeal carcinoma (NPC) and to evaluate the prognostic significance of BRMS1 in NPC patients.

**Methods:**

BRMS1 expression was examined in NPC cell lines using quantitative reverse transcription-polymerase chain reaction and Western blotting. NPC cells stably expressing BRMS1 were used to perform wound healing and invasion assays in vitro and a murine xenograft assay in vivo. Immunohistochemical staining was performed in 274 paraffin-embedded NPC specimens divided into a training set (n = 120) and a testing set (n = 154).

**Results:**

BRMS1 expression was down-regulated in NPC cell lines. Overexpression of BRMS1 significantly reversed the metastatic phenotype of NPC cells in vitro and in vivo. Importantly, low BRMS1 expression was associated with poor distant metastasis-free survival (DMFS, *P* < 0.001) and poor overall survival (OS, *P* < 0.001) in the training set; these results were validated in the testing set and overall patient population. Cox regression analysis demonstrated that low BRMS1 expression was an independent prognostic factor for DMFS and OS in NPC.

**Conclusions:**

Low expression of the metastasis suppressor BRMS1 may be an independent prognostic factor for poor prognosis in NPC patients.

## Background

According to the Global Cancer Statistics released by the International Agency for Research on Cancer (IARC), there were an estimated 84,400 incident cases of nasopharyngeal carcinoma (NPC) and 51,600 NPC-related deaths in 2008 [1]. Highly metastatic NPC is one of the most common malignant tumors in southern China, especially in the province of Guangdong, [2]. For patients with locally advanced disease receiving chemoradiation therapy, the rate of distant metastasis may range between 13 and 21% [3,4]. Studies by our group and others have shown that although the increasingly prevalent use of intensity-modulated radiation therapy (IMRT) and concurrent chemoradiation therapy for locoregionally advanced NPC has improved local and regional control in NPC, distant metastasis has become a predominant pattern of treatment failure in NPC patients who do not present metastases at diagnosis [5,6].

Metastasis is a multistep process in which cancer cells disseminate and establish secondary tumors at sites distant from the primary tumor; metastasis is estimated to be responsible for approximately 90% of all cancer deaths [7]. The breast cancer metastasis suppressor 1 (BRMS1) gene was originally identified as a true metastasis suppressor gene in breast cancer cell lines as stable overexpression of BRMS1 suppressed pulmonary metastasis but did not inhibit primary tumor growth [8]. Subsequent studies have demonstrated that BRMS1 dramatically suppresses the metastatic phenotype in vitro in cells from several othertypes of cancer, including melanoma [9,10], ovarian cancer [11], bladder cancer [12] and lung cancer [13]. BRMS1 was also shown to inhibit metastasis in xenograft models of breast cancer [8], melanoma [9] and ovarian carcinoma [11]. However, there are no published reports on in vitro or in vivo BRMS1 expression and function in NPC and no available articles that address a possible relationship between BRMS1 expression and clinical outcomes in NPC.

In this study, we aimed to examine BRMS1 expression and functional status in vitro and in vivo and to explore its clinical significance in clinical NPC specimens. The results may help to understand the mechanisms of metastasis in NPC and provide information for development of personalized therapies for NPC patients with distant metastasis.

## Methods

### Study design

Results of qRT-PCR and Western blotting indicated that BRMS1 expression was low in NPC cells. Therefore we created cell lines stably overexpressing *BRMS1* or the corresponding empty vector. The effect of BRMS1 on migration and invasion was observed in artificial wound healing assay, transwell invasion array in vitro. A murine model of NPC was constructed to further investigate the metastasis-inhibiting effects of BRMS1 compared with vector (n = 8 per group). To determine the clinical significance of BRMS1 in NPC patients, we detected BRMS1 expression by immunohistochemistry in 274 tumor specimens which were randomly divided into a training set (n = 120) and a testing set (n = 154). The association between BRMS1 expression and patient outcomes was explored in the training set and then validated in the testing set and overall patient population.

### Cell lines and cell culture

The NPC cell lines (SUNE-1, CNE-1, C666-1, CNE-2 and HNE-1) and the immortalized nasopharyngeal epithelial cell line (NP69) were the kind gifts of Professor Zeng Mu-sheng at Sun Yat-sen University Cancer Center (SYSUCC). All NPC cell lines were maintained in RPMI 1640 (Invitrogen, Beijing, China) supplemented with 10% fetal bovine serum (Gibco, Montevideo, Uruguay), while the NP69 cells were cultured in Keratinocyte-SFM (Invitrogen, Auckland, NZ) supplemented with bovine pituitary extract, as previously described [14]. All the cell lines were incubated at 37°C in a 5% CO_2_ incubator.

### RNA extraction and qRT-PCR

The total RNA was extracted from the cell lines using TRIzol reagent (Life Technologies, Grand Island, NY). The first-strand cDNA was synthesized using the M-MLV First-strand Synthesis Kit (Invitrogen, China). The following PCR primers were used for *BRMS1* and glyceraldehyde-3-phosphate dehydrogenase (GAPDH): BRMS1 forward, 5^′^-AAGGCACCTCTGGTTTCTGG-3^′^; *BRMS1* reverse, 5^′^-TGTGAACAGCAGGGTCAAGGT-3^′^; *GAPDH* forward, 5^′^-CTCCTCCTGTTCG ACAGTCAGC-3^′^ and *GAPDH* reverse, 5^′^-CCCAATACGACCAAATCCGTT-3^′^. The quantitative PCR was performed using SYBR Green qPCR SuperMix-UDG reagent (Invitrogen, China) and an ABI PRISM 7900HT sequence detection system (Applied Biosystems, USA). The *BRMS1* cycle threshold (Ct) was normalized to the *GAPDH* internal reference.

### Western blotting

The protein was extracted as previously described [14] , loaded onto 12% SDS-PAGE gels and transferred to PVDF membranes. The membranes were blocked with a mouse anti-human BRMS1 antibody (1:500; Abnova, Taipei, Taiwan). BRMS1 expression was detected with horseradish peroxidase-conjugated goat anti-mouse antibody (1:10,000, Merck, Darmstadt, Germany) and a Super Signal enhanced chemiluminescence substrate (Pierce, Rockford, IL, USA). A rabbit anti-human α-tubulin antibody (1:1000, CST, USA) was used to confirm equal loading.

### Establishing NPC cells that stably expressed BRMS1

Following the manufacturer’s instructions, the CNE-2 and SUNE-1 cell lines were stably transfected using a Lenti-Pac™ HIV Expression Packaging Kit (GeneCopoeia, Rockville, MD, USA) and a plasmid encoding *BRMS1* or a control vector plasmid. Briefly, EndoFectin Lenti reagent was used to transfect the parental CNE-2 and SUNE-1 cells with 2.5 μg of either a lentiviral *BRMS1* ORF plasmid (EX-V1241-Lv105, GeneCopoeia) or a control vector plasmid (EX-NEG-Lv105, GeneCopoeia). The cells were allowed to grow under puromycin (0.5 μg/ml) selection for 10 days. Western blotting and qRT-PCR were used to analyze the BRMS1 expression. The cells overexpressing *BRMS1* were renamed CNE-2B and SUNE-1B, and the vector control cells were renamed CNE-2V and SUNE-1V.

### Wound healing assay

The CNE-2B/V and SUNE-1B/V cells were seeded in 6-well cell culture plates. When the cell confluence reached approximately 90%, the cells were serum-starved for 24 h, and wounds were then created by scraping the cell monolayer with a 200-μl pipette tip. The cells were then rinsed with serum-free medium to remove floating cells and debris. The culture plates were incubated at 37°C. The width of the wounds was measured at various times. Representative wounds were photographed under a phase-contrast inverted microscope (4× objective, Leica, Wetzlar, Germany). The experiment was repeated three times.

### Transwell invasion assays

The log phase CNE-2B/V and SUNE-1B/V cells were trypsinized and suspended in single cell solutions. A total of 1 × 10^5^ cells in 200 μl serum-free RPMI 1640 medium were seeded on 8-μm-pore polycarbonate membrane chambers in Transwell plates (Corning, Corning, NY, USA) that were coated Matrigel (BD Biosciences, San Jose, CA), and 600 μl of RPMI 1640 containing 20% FBS was added to the lower chamber. After incubation for 18 hours at 37°C in a 5% CO_2_ incubator, the cells on the top insert surface were removed by wiping with a cotton swab. The cells that had invaded to the bottom surface of the insert were fixed with a 3:1 mixture of methanol and acetic acid for 10 minutes, stained in 0.5% crystal violet for 30 minutes, rinsed in PBS and then subjected to microscopic inspection (200×). The numbers of invading cells were obtained by counting the number of cells in five random microscopic fields per membrane. The experiment was repeated three times.

### In vivo lung metastasis model

Male BALB/c nude mice between 5 and 6 weeks old were purchased from the Hunan Slac Jingda Laboratory Animal Co., Ltd. (Changsha, Hunan province, China) and were in quarantined for a week before treatment. Animals were provided with standard laboratory chow and tap water ad libitum under special pathogen free (SPF) conditions. All the animal studies were conducted in accordance with "Animal Research: Reporting In Vivo Experiments" (ARRIVE) guidelines and the guidelines of Institutional Animal Care and Use Committee at SYSUCC. All mice were treated humanely throughout the experimental period.

To assay for lung metastases, 1× 10^6^ SUNE-1B/V cells in 200 μl PBS were injected into the lateral tail veins of the mice (n =8 per group). Nine weeks later, the mice were necropsied after anesthesia. The lungs of mice were fixed in 3.7% formaldehyde, 5% glacial acetic acid, and 72% ethanol for at least one day before proceeding to paraffin embedding. Serial 5-μm sections were cut, and one of every ten slides was stained with H&E for histopathological examination.

### Tissue specimens, patient information and follow-up

274 biopsy-proven and non-distant-metastasis paraffin-embedded NPC samples and 8 noncancerous nasopharyngeal (NNP) tissues were collected at SYSUCC between April 2003 and December 2006. None of the NPC patients received any therapies before biopsies. Prior informed consents from the patients and approval from the medical ethics committee of SYSUCC were obtained. The patients’ clinical information is summarized in Table 1. The clinical staging of all the NPC patients was re-performed according to the 7^th^ International Union Against Cancer (UICC)/American Joint Committee on Cancer (AJCC) system [15]. The 274 NPC FFPE specimens were randomly divided into a training set and a testing set using a random number table generated by SPSS 16.0 software (SPSS, Chicago, IL, USA). 

**Table 1:** The correlations between BRMS1 expression and the clinicopathological characteristics of nasopharyngeal carcinoma

**Characteristic**	**Training set (n=120)**			**Testing set (n=154)**			**Overall patients (n=274)**		
	**low**	**high**	P^*^	**low**	**high**	P^*^	**low**	**high**	P^*^
	**expression**	**expression**		**expression**	**expression**		**expression**	**expression**	
	**no. (%)**	**no. (%)**		**no. (%)**	**no. (%)**		**no. (%)**	**no. (%)**	
Gender									
Male	28 (66.7)	60 (76.9)	0.226	44 (77.2)	74 (76.3)	0.898	72 (72.7)	134 (76.6)	0.286
Female	14 (33.3)	18 (23.1)		13 (22.8)	23 (23.7)		27 (27.3)	41 (23.4)	
Age (years)									
≤ 46	20 (47.6)	37 (47.4)	0.985	31 (54.4)	49 (50.5)	0.643	51 (51.5)	86 (49.1)	0.401
> 46	22 (52.4)	41 (52.6)		26 (45.6)	48 (49.5)		48 (49.5)	89 (50.9)	
WHO type									
Type III	40 (95.2)	74 (94.9)	1.000	56 (98.2)	94 (96.9)	1.000	96 (97.0)	168 (96.0)	0.482
Other type	2 (4.8)	4 (5.1)		1 (1.8)	3 (3.1)		3 (3.0)	7 (4.0)	
VCA-IgA									
≥ 1:320	23(54.8)	41(52.6)	0.818	28(49.1)	48(49.5)	0.965	51(51.5)	89(50.9)	0.917
< 1:320	19(45.2)	37(47.4)		29(50.9)	49(50.5)		48(48.5)	86(49.1)	
EA-IgA									
≥ 1:20	20(47.6)	43(55.1)	0.432	33(57.9)	50(51.5)	0.445	53(53.5)	93(53.1)	0.950
< 1:20	22(52.4)	35(44.9)		24(42.1)	47(48.5)		46(46.7)	82(46.9)	
AER									
≥ 63%	8(19.0)	23(29.5)	0.213	17(29.8)	26(26.8)	0.687	25(25.3)	49(28.0)	0.623
< 63%	34(81.0)	55(70.5)		40(70.2)	71(73.2)		74(74.7)	126(72.0)	
UICC 7^th^ T stage									
T1	5 (11.9)	16 (20.5)	0.470	11 (19.3)	18 (18.6)	0.996	16 (16.2)	34 (19.4)	0.853
T2	17 (40.5)	22 (28.2)		18 (31.6)	32 (33.0)		35 (35.4)	54 (30.9)	
T3	9 (21.4)	19 (24.4)		16 (28.1)	26 (26.8)		25 (25.3)	45 (25.5)	
T4	11 (26.2)	21 (26.9)		12 (21.1)	21 (21.6)		23 (23.2)	42 (23.7)	
UICC 7^th^ N stage									
N0	4 (9.5)	14 (17.9)	0.644	8 (14.0)	14 (14.4)	0.837	12 (12.1)	28 (16.0)	0.732
N1	22 (52.4)	36 (46.2)		24 (42.1)	47 (48.5)		46 (46.5)	83 (47.4)	
N2	10 (23.8)	16 (20.5)		15 (26.3)	20 (20.6)		25 (25.3)	36 (20.6)	
N3	6 (14.3)	12 (15.4)		10 (17.5)	16 (16.5)		16 (16.2)	28 (16.0)	
									
No	4 (9.5)	14 (17.9)	0.307	8 (14.0)	14 (14.4)	0.806	12 (12.1)	28 (16.0)	0.382
Yes	38 (90.5)	64 (82.1)		49 (86.0)	83 (85.6)		87 (87.9)	147 (84.0)	
									
I	0 (0.0)	4 (5.1)	0.513	1 (1.8)	3 (3.1)	0.949	8 (2.9)	8 (2.9)	0.538
II	13 (31.0)	22 (28.2)		17 (29.8)	27 (27.8)		79 (28.8)	79 (28.8)	
III	13 (31.0)	22 (28.2)		19 (33.3)	31 (32.0)		85 (31.0)	85 (31.0)	
IV	16 (38.0)	30 (38.5)		20 (35.1)	36 (37.1)		102 (37.2)	102 (37.2)	
									
No	24 (57.1)	67 (85.9)	**0.000**	35 (61.4)	83 (85.6)	**0.000**	59 (59.6)	150 (85.7)	**0.000**
Yes	18 (42.8)	11 (14.1)		22 (38.6)	14 (14.4)		40 (40.4)	25 (14.3)	

* *p* value was calculated using the chi-squared or Fisher's exact tests. ^#:^ The presence of distant metastasis on or before the last follow-up (April 30, 2011).

After completing their therapy, the patients returned for follow-up appointments every 3 months for the first 2 years and every 6 months thereafter. The last follow-up date was April 30, 2011, and the median follow-up period was 61.9 months (range, 3.1-85.4 months). All the events were measured from the date of diagnosis. The following end points were assessed (as the time to the defining event): distant metastasis-free survival (DMFS, with “distant” defined as metastasis to other organs or tissues) and overall survival (OS, with “overall” defined as death due to any cause).

### Immunohistochemistry

Immunohistochemical staining was performed similarly to previous report [14]. The Mouse anti-BRMS1 antibody (1:400; Abnova, Taiwan) and biotinylated anti-mouse secondary antibody (zsbio, Beijing, China) were used. The degree of immunostaining was reviewed and scored independently by two pathologists. The staining intensity was scored as 0 (negative), 1 (weak), 2 (medium), and 3 (strong). Extent of staining was scored as 0 (0%), 1 (1% to 25%), 2 (26% to 50%), 3 (51% to 75%), and 4 (76 to 100%) according to the percentages of positive tumor cells in the tumor area. The final staining score was the sum of the intensity and extent score [16].

### Statistical analysis

The data were expressed as the mean ± SD. An independent-sample T test was used to test for significant differences between continuous variables. The distributions of the NPC patients’ clinical parameters were compared between the high and low BRMS1 expression groups using the chi-square or Fisher’s exact tests. Kaplan-Meier survival analysis was used to compare the patient survival times. The log-rank test was used to evaluate the differences in survival probabilities between the groups. A Cox proportional hazards regression analysis with backward stepwise selection was used to explore the independent predictive factors for DMFS and OS. All the quoted *p* values are two-sided, and *P* < 0.05 was considered to be statistically significant. The statistical analyses were performed using SPSS 16.0 (SPSS, Chicago, IL).

## Results

### BRMS1 expression is decreased in NPC cells

**Figure 1:**
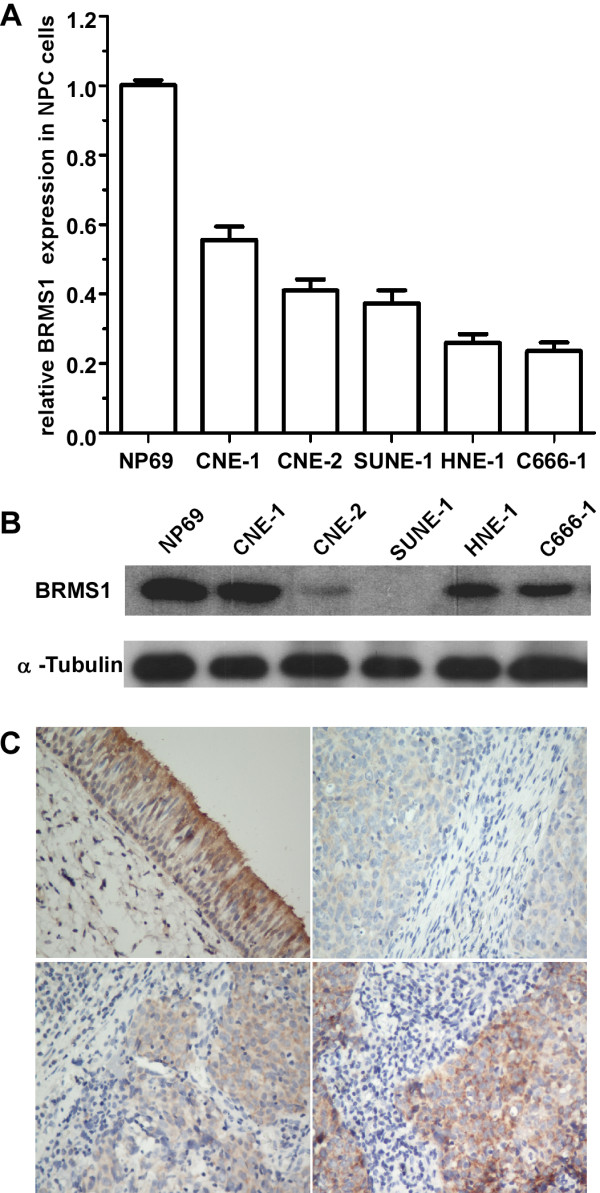
**BRMS1 expression in NPC cells and tissues.** Quantitative RT-PCR (**A**) and western blotting analysis (**B**) of BRMS1 mRNA and protein expression in parental NPC cells. Quantitative RT-PCR analysis of BRMS1 mRNA was normalized to glyceraldehyde 3-phosphate dehydrogenase (GAPDH). The quantitative RT-PCR data are represented as the mean ± SD (* *p* < 0.01; Student’s t-test). Equal protein loading was determined by α-tubulin in western blotting. (**C**) analysis of BRMS1 protein expression in NPC and noncancerous nasopharyngeal (NNP) tissues using immunohistochemistry ( IHC). The NNP tissue (upper left) showed strong BRMS1 staining. The NPC tissues showed various degrees of BRMS1 staining: weak (upper right), moderate (lower left) and strong (lower right) (magnification, 100×).

Since BRMS1 is a metastasis suppressor in many types of cancer, we hypothesize that NPC progression, especially NPC metastasis, may be related to BRMS1 levels. The results of quantitative reverse transcription-polymerase chain reaction (qRT-PCR, Figure 1A) and western blotting (Figure 1B) indicated that both BRMS1 mRNA and protein were markedly decreased in the examined NPC cell lines compared to the NP69, suggesting that low BRMS1 expression may be involved in NPC progression.

### BRMS1 inhibits migration and invasion in vitro

**Figure 2:**
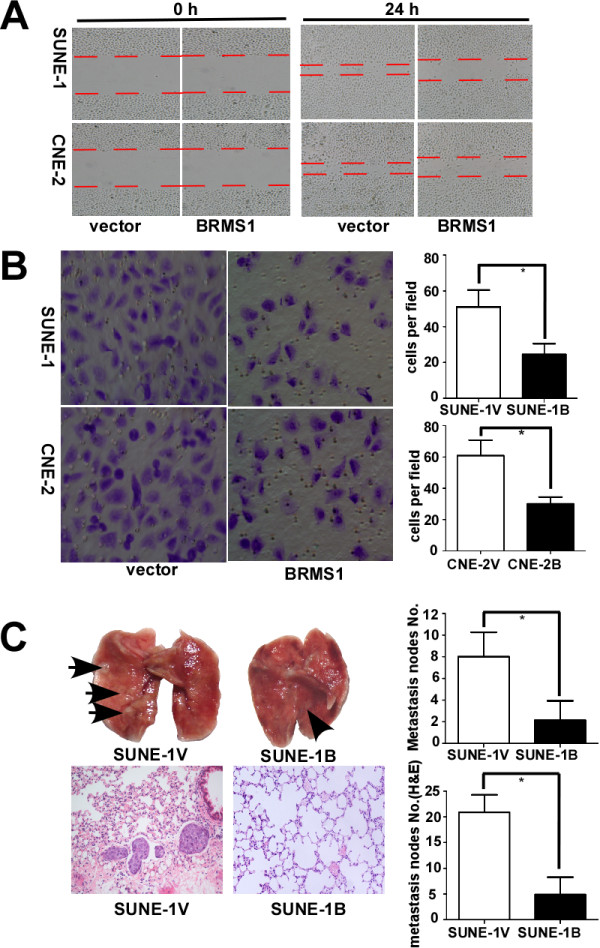
**BRMS1 inhibits NPC cell migration and invasion in vitro and suppresses lung metastasis in vivo.** (**A**) The effect of BRMS1 overexpression on cell migration in the wound healing assay. The right panels show a slower wound healing response in the BRMS1-overexpressing cells than in the vector control cells 24 h after scratching (magnification, 40×). (**B**) The effect of BRMS1 overexpression on cell invasion in the Transwell migration assay. The left panels are representative photomicrographs (magnification, 100×), while the right panels are the numbers of trans-membrane cells per field (magnification, 100×) counted in five random fields for each of the BRMS1-overexpressing and control groups in triplicate parallel experiments. (* *p* < 0.01; Student’s *t*-test). (**C**) BRMS1 overexpression inhibits metastasis in murine NPC xenografts. Mouse tumor xenografts were created (n = 8 per group). Representative macroscopic photographs of pulmonary metastases; the arrowheads indicate the metastatic nodules on the surface of the lungs (upper left in C). The average numbers of metastases in each group are shown in parallel on the right. Lung sections from each group were stained with hematoxylin and eosin (H&E) to quantify the degree of lung metastasis (lower left in C). Histograms depicting the average number of microscopic metastases in each group are shown on the right. (* *p* < 0.01; Student’s t-test).

To confirm that low BRMS1 expression may cause NPC cell metastasis, we conducted an in vitro cell migration and invasion assay using the CNE-2B/V and SUNE-1B/V cell lines that stably expressed BRMS1 or an empty vector. The qRT-PCR and western blot analyses confirmed that BRMS1 mRNA and protein expression were greater in the CNE-2B and SUNE-1B cells than in the corresponding CNE-2V and SUNE-1V control cells (data not shown). The effect of BRMS1 on NPC cell migration was then examined in the wound healing and migration assays. As shown in Figure 2A, the wounded BRMS1-expressing cells (CNE-2B and SUNE-1B) traveled a significantly shorter distance after a 24 h incubation period than did the corresponding vector control cells. In addition, a Transwell invasion assay was conducted to explore the effects of BRMS1 on NPC cell invasion. The results demonstrated that the invasive abilities of the CNE-2B and SUNE-1B cells were significantly lower than those of the corresponding vector cells, indicating massive transmembrane invasion (*P* < 0.01 respectively, Figure 2B). These results were further quantified by calculating the number of invading cells per field of high magnification.

### BRMS1 inhibits pulmonary metastasis in vivo

To define the function of BRMS1, a murine xenograft model of distant NPC metastasis was constructed. Upper-left of Figure 2C showed that there were few metastatic nodules on the surface of excised lungs in the SUNE-1B group, whereas there were many in the control cohort (upper-middle of Figure - 2C). This macroscopic observation is quantified in the associated bar graphs, demonstrating a significant decrease in the number of metastatic nodules in the presence of BRMS1 (*p* < 0.01, upper-right of Figure - 2C).

In addition, lung sections from each group were stained with H&E to show the degree of metastasis. The results of the hematoxylin and eosin (H&E) staining indicated significantly fewer lung metastasis nodules in the SUNE-1B group than in the SUNE-1V group (4.9 ± 3.4 vs. 20.8 ± 3.4, *p* < 0.01, lower of Figure 2C). These results suggested that BRMS1 expression significantly inhibited xenograft cell invasion in the surrounding tissue. These results above indicated the metastasis-inhibiting role of BRMS1 in this xenograft model.

### BRMS1 protein expression is decreased in NPC tissues

To explore the association between BRMS1 expression and clinical outcome in NPC patients, immunohistochemistry staining was performed. Representative photomicrographs depicting the BRMS1 staining are shown in Figure 1C. The BRMS1 protein expression was lower in the NPC tissues than in the NNP tissue controls (Figure 1C).

### The relationship between BRMS1 expression and clinicopathological features in NPC patients

A receiver operating characteristic (ROC) curve analysis was used to select the cutoff scores for BRMS1 expression in the training set, as previously described [17]. The best cutoff scores of BRMS1, viral capsid antigen immunoglobulin A (VCA-IgA), early antigen immunoglobulin A (EA-IgA) and antienzyme rate (AER) of Epstein-Barr virus (EBV) DNase-specific neutralizing antibody for DMFS were 4, 1:320, 1:20 and 63% respectively (Table 1Additional file 1: Figure S1).

The clinicopathological features of these two cohorts and the overall patient set, including patient age and gender, WHO pathology type, VCA, EA, AER, tumor stage, node stage, clinical stage and metastasis, are summarized and stratified according to BRMS1 expression in Table 1. We found that in the training set, more patients developed metastasis in the low BRMS1 expression group than in the high BRMS1 expression group (42.8% vs. 14.1%, *p <* 0.001). Similar results were observed in the testing set (38.6% vs. 14.1%, *p <* 0.001) and overall patient set (40.4% vs. 14.3%, *p <* 0.001). No significant associations were found between BRMS1 expression and any of the other clinicopathological features in any set of patients.

### Low BRMS1 expression is associated with poor DMFS and OS in NPC patients

**Figure 3:**
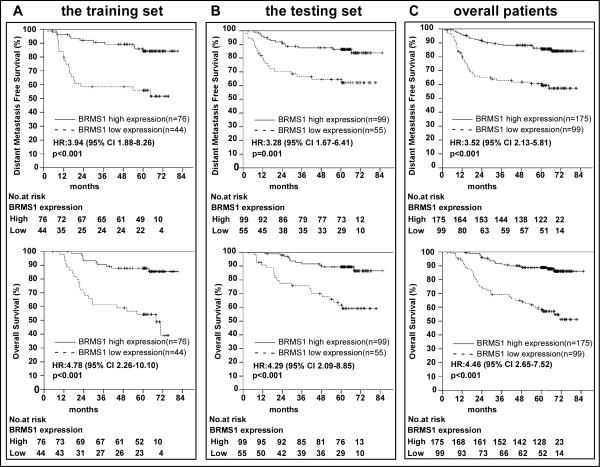
**Survival analysis of BRMS1 expression in the training set, testing set and overall patient population.** (**A**) Low BRMS1 expression was correlated with poor distant metastasis-free survival (upper in A) and overall survival (lower in A) in the training cohort. These results were validated in the testing cohort (**B**) and overall patient population (**C**). HR: hazard ratio; CI: confidence interval. The HR values were calculated using a univariate Cox regression analysis, and the *p* values were calculated using the log-rank test.

The median follow-up time for the entire patient set was 61.8 months. The cumulative 5-year survival rate was only 52.53% (95% confidence interval (CI), 42.69% - 62.36%) in the low BRMS1 expression group, whereas it was 74.28% (95% CI, 65.41% - 81.87%) in the high BRMS1 expression group. In the training set the patients with low BRMS1 expression had poorer DMFS (upper of Figure 3A) and poorer OS (lower of Figure 3A) than those with high BRMS1 expression (hazard ratio (HR) 3.94, 95% CI, 1.88-8.26, *p* < 0.001 for DMFS; HR, 4.78, 95% CI, 2.26-10.10, *p* < 0.001 for OS). These results were validated in the testing (Figure 3B) and overall patient sets (Figure 3C).

### BRMS1 is an independent prognostic factor for DMFS and OS

A multivariate Cox regression analysis was performed to explore the effect of various factors on DMFS and OS. Low BRMS1 expression was indeed found to be a significant independent prognostic factor for poor DMFS (HR, 4,54, 95% CI, -2.11 – 9.76, *p* < 0.001, Table 2) and OS (HR, 6.56, 95% CI, 2.97 – 14.41, *p* < 0.001, Table 2) in the training set. Similar results were also observed in the testing set (HR, 3.31, 95% CI: 1.69 – 6.47, *p* < 0.001 for DMFS and HR, 4.39, 95% CI, 2.14– 9.01, *p* < 0.001 for OS, Table 2) and overall patient population (HR, 3.59, 95% CI, 2.17–5.92, *p* < 0.001 for DMFS and HR, 4.77, 95% CI, 2.82–8.06 , *p* < 0.001 for OS, Table 2). In addition, clinical stage was found to be an independent prognostic factor for DMFS and OS in the training, testing and overall patient sets; AER was only an independent prognosis factor in the overall patient population (Table 2). These results indicated that BRMS1 is an independent prognostic factor for DMFS and OS in NPC patients.

**Table 2:** **A multivariable Cox regression analysis**^**# **^**of BRMS1 expression and survival in nasopharyngeal carcinoma**

	**For distant metastasis free survival**			**For overall survival**		
	**HR**	**95% CI**	P*	**HR**	**95% CI**	P*
**Training set**						
sex (men vs. women)	0.51	0.20-1.27	0.15	0.29	0.11-0.79	0.11
age (> 46 vs. ≤ 46)	1.16	0.55-2.46	0.54	1.11	0.53-2.35	0.72
WHO type (type I vs. type II)	0.89	0.20-3.99	0.80	1.31	0.26-5.02	0.81
TNM stage (III, IV vs. I, II)	3.91	1.35-11.29	**0.01**	2.59	0.99-6.83	**0.04**
BRMS1 (low vs. high)	4.54	2.11-9.76	**0.00**	6.56	2.97-14.41	**0.00**
VCA-IgA (≥ 1:320 vs. < 1:320)	0.73	0.26-2.03	0.93	0.41	0.15-1.13	0.21
EA-IgA (≥ 1:20 vs. < 1:20)	1.44	0.52-4.01	0.76	1.80	0.66-4.92	0.88
AER (≥ 63% vs. < 63%)	0.73	0.36-1.47	0.35	1.51	0.74- 2.51	0.07
**Testing set**						
sex (men vs. women)	0.56	0.22-1.38	0.28	0.76	0.36-1.80	0.62
age (> 46 vs. ≤ 46)	1.25	0.65-2.43	0.47	1.54	0.77-3.10	0.14
WHO type (type I vs. type II)	1.05	0.13-8.39	0.60	0.67	0.08-5.60	0.42
TNM stage (III, IV vs. I, II)	3.19	1.24-8.21	**0.02**	3.91	1.38-11.11	**0.01**
BRMS1 (low vs. high)	3.31	1.69-6.47	**0.00**	4.39	2.14-9.01	**0.00**
VCA-IgA (≥ 1:320 vs. < 1:320)	0.47	0.19-1.15	0.97	0.44	0.18-1.10	0.71
EA-IgA (≥ 1:20 vs. < 1:20)	2.78	1.12-6.94	0.19	2.33	0.91-5.98	0.31
AER (≥ 63% vs. < 63%)	0.57	0.26-1.29	0.32	1.03	0.49-2.19	0.71
**Overall patient population**						
sex (men vs. women)	0.63	0.34-1.18	0.18	0.57	0.30-1.07	0.15
age (> 46 vs. ≤ 46)	1.18	0.72-1.92	0.38	1.32	0.80-2.18	0.25
WHO type (type I vs. type II)	0.90	0.28-2.91	0.74	0.89	0.27-2.88	0.57
TNM stage (III, IV vs. I, II)	3.39	1.68-6.86	**0.00**	3.24	1.60-6.57	**0.00**
BRMS1 (low vs. high)	3.59	2.17-5.92	**0.00**	4.77	2.82-8.06	**0.00**
VCA-IgA (≥ 1:320 vs. < 1:320)	0.62	0.31-1.21	0.84	0.53	0.27-1.04	0.29
EA-IgA (≥ 1:20 vs. < 1:20)	1.91	0.95-3.85	0.16	1.64	0.82-3.29	0.72
AER (≥ 63% vs. < 63%)	1.20	0.70-2.07	0.36	1.69	1.00-2.86	**0.04**

# The variables were selected using the backward Wald stepwise selection method. * The *p* values were calculated using an adjusted Cox regression model. The following parameters were included as covariates in each of these models: sex (men vs. women), age (> 46 vs. ≤ 46), WHO type (type I vs. type II), TNM stage (III and IV vs. I and II), BRMS1 expression (low vs. high) VCA-IgA (≥ 1:320 vs. < 1:320), EA-IgA (≥ 1:20 vs. < 1:20) and AER (≥ 63% vs. < 63%). VCA-IgA: viral capsid antigenimmunoglobulin A; EA-IgA: early antigenimmunoglobulin A; AER: antienzyme rate (AER) of Epstein-Barr virus (EBV) DNase-specific neutralizing antibody; HR: hazard ratio; CI: confidence interval.

### BRMS1 is superior to EBV biomarkers for predicting DMFS and OS

The area under ROC curve (AUROC) of BRMS1 for predicting DMFS is bigger than that of VCA, EA and AER respectively in the training set ( Additional file 1: Figure S1). Similar trends were found in the testing set and overall patient population (data not shown). These indicated that BRMS1 had a higher accuracy for predicting DMFS than all of the tested EBV biomarkers. Moreover, BRMS1 demonstrated a higher ability to predict OS than VCA, EA and AER in the three cohorts (data not shown). As described above, multivariate Cox regression analysis indicated that BRMS1 expression was an independent factor for both DMFS and OS in the training set, testing set and overall patient population; however, VCA and EA were not any prognostic value in any group and AER was only an independent factor of OS in the overall patient population. Therefore, BRMS1 is superior to EBV biomarkers for predicting DMFS and OS.

## Discussion

In this study, we found low BRMS1 expression in the NPC cell lines and tissue specimens. Artificial overexpression of BRMS1 in the NPC cell lines suppressed migration and invasion in vitro and inhibited the formation of pulmonary metastases in vivo in the nude mice. Furthermore, low BRMS1 expression was significantly associated with poor DMFS and OS in the NPC patients. These results suggested that low BRMS1 expression may play important roles in NPC metastatic process. To our knowledge, this is the first study to reveal a correlation between BRMS1 expression and clinical metastasis and survival in NPC patients.

BRMS1 has previously been demonstrated to be a metastasis-suppressing gene in breast cancer [18,19], melanoma [10,20], ovarian cancer [11], bladder cancer [12] and lung cancer [13,21]. Low BRMS1 expression levels have been detected in cell lines [8,9,11-13,22] and tissue specimens [11,13,19] from various human cancers. Our study found that BRMS1 expression levels were markedly lower in the NPC cell lines and tissues than in the NP69 and NNP tissues, indicating that downregulation of BRMS1 may play an important role in NPC progression.

To explore the effect of BRMS1 on NPC metastasis, we firstly created NPC cell lines that stably expressed BRMS1 or an empty vector. Increased BRMS1 expression was shown to decrease NPC cells migration and invasiveness in vitro compared to the corresponding control vector. To further define the metastasis-suppressing function of BRMS1, a nude mouse model of NPC metastasis was constructed. The macroscopic and microscopic observations of the metastatic mouse lung nodules indicated that BRMS1 significantly suppressed pulmonary metastasis formation in vivo. All of the functional studies above demonstrate the inhibitory effects of BRMS1 on NPC metastasis, which is consistent with reports on its effects in other cancers. In breast cancer, BRMS1 expression was higher in the neo11/435 metastasis-suppressed hybrid cell line than in the MDA-MB-435 parental line, which is a highly metastatic breast cancer cell line in vitro. In vivo, ectopic BRMS1-expressing MDA-MB-435 cells showed significantly decreased incidence and number of lung and regional lymph node metastases when the cells were orthotopically injected [8]. BRMS1 expression has also been shown to be lower in a highly metastatic human bladder carcinoma cell line (T24T) than in the less metastatic T24 parental cell line. In vivo modeling found that mice inoculated with T24T cells had significantly more metastases than mice inoculated with T24 cells [12]. Similar results were obtained for lung cancer [13], ovarian cancer [9] and melanoma [10,20].

In this study, we further investigated the clinical significance of BRMS1 in NPC patients using immunohistochemistry (IHC) to assay BRMS1 expression in 274 NPC tissue specimens. BRMS1 was hypothesized to be associated with lymph node metastasis, which is usually correlated with distant metastasis. In our study, however, BRMS1 expression did not correlate with nodal status (Table 1). This result was consistent with previous breast [19,23] and lung cancer [13] studies and may imply that BRMS1 plays different roles in lymph node and distant metastases. Li et al. demonstrated that BRMS1 inhibits blood vessel formation in nude mice [10] by regulating ING4 [24]. As expected, the patients with low BRMS1 expression experienced more metastasis during the follow-up period compared to those with high BRMS1 expression, suggesting that BRMS1 can suppress cancer metastasis in NPC patients. Our results were consistent with those obtained for other neoplasms, such as breast cancer [25], melanoma [10] and NSCLC [13]. However, there is some disagreement on the role of BRMS1 in cancer. Lombardi et al. found that higher BRMS1 mRNA expression was associated with poor disease-free and overall survival [26], which is inconsistent with the results of other studies.

In our study, a multivariate Cox regression analysis demonstrated that both TNM stage and BRMS1 expression were independent prognostic factors for DMFS and OS in the training set, and this conclusion was validated in the testing and overall patient sets. Currently, the current TNM staging system is useful for predicting NPC outcome [15]. However, patients with identical disease stages who receive similar treatments often display considerable variability in their clinical outcomes, indicating that the TNM stage is still far from a perfect predictor. EBV biomarkers such as VCA, EA and AER have been widely used in the diagnosis of NPC, but are not appropriate in predicting prognosis. In our study, BRMS1 expression was an independent prognostic factor for both DMFS and OS in the training set, testing set and overall patient population, while VCA and EA had no prognostic value in any group and AER was only an independent factor of OS in the overall patient population. Therefore, BRMS1 levels used in conjunction with the TNM staging system have the potential to more effectively predict patient metastasis potential and prognosis and could guide the development of more personalized therapies for this disease.

Although the clinical significance of BRMS1 in many cancers has been established, the mechanisms by which BRMS1 expression is decreased in tumors is still not clear. Some studies have shown that the BRMS1 promoter is methylated in many cancers, which might contribute to low BRMS1 expression. Metge et al. discovered a CpG island (−3477 to −2214) in the BRMS1 promoter which is hypermethylated across several breast cancer cell lines [27]. Another study demonstrated BRMS1 promoter methylation of in lung cancer [21]. A recent study showed that phosphorylation of RelA/p65 promotes DNMT-1 recruitment to chromatin following BRMS1 promoter methylation and transcriptional repression [22]. BRMS1 has also been reported to interact with the mSin3 chromatin remodeling complex and to recruit histone deacetylases to suppress downstream gene expression [28]. It has been shown that BRMS1 physically interacts with the RelA/p65 subunit of NF-kB and inhibits IkBa phosphorylation, thus negatively regulating the NF-kB pathway [29,30]. Several metastasis-related genes, such as epidermal growth factor receptor [31] and osteopontin [32], have been reported to be regulated by BRMS1.

## Conclusion

In summary, this is the first study to demonstrate that reduced BRMS1 expression is associated with metastasis and survival in NPC patients. We found that BRMS1 suppressed NPC metastasis in vitro and in vivo. A survival analysis showed that BRMS1 expression was an independent predictive factor for DMFS and OS in NPC patients. And BRMS1 was better than EBV biomarkers in predicting DMFS and OS. The results of this study suggest that BRMS1 is a potential biomarker for metastasis and prognosis in NPC patients and may provide a basis for developing gene therapy to prevent or treat NPC metastasis.

## Abbreviations

NPC: Nasopharyngeal carcinoma; BRMS1: Breast cancer metastasis suppressor 1; qRT-PCR: Quantitative reverse transcription-polymerase chain reaction; ROC: Receiver operating characteristic; H&E: Hematoxylin and eosin; NNP: Noncancerous nasopharyngeal; DMFS: Distant metastasis-free survival; OS: Overall survival.

## Competing interests

The author(s) declare that they have no competing interests.

## Authors’ contributions

RXC, NL, BJH and QMH were involved in the study design, performed the experiments, and drafted the manuscript. WFL, SY, TLL, CM, and JN participated in sample collection. CL and GY performed the statistical analysis. JPY and JZ reviewed the manuscript. JM and HYW conceived the idea for the study, contributed to the overall experiment design and revised the manuscript. All authors read and approved the final manuscript.

## Pre-publication history

The pre-publication history for this paper can be accessed here:

http://www.biomedcentral.com/1471-2407/12/376/prepub

## Supplementary Material

Additional file 1 Figure S1. Click here for file
