# Supplementary figures and images for: Low BRMS1 expression promotes nasopharyngeal carcinoma metastasis in vitro and in vivo and is associated with poor patient survival
Source: BMC Cancer. 2012 Aug 29;12:376. doi: 10.1186/1471-2407-12-376 (PMC3517767; doi:10.1186/1471-2407-12-376)

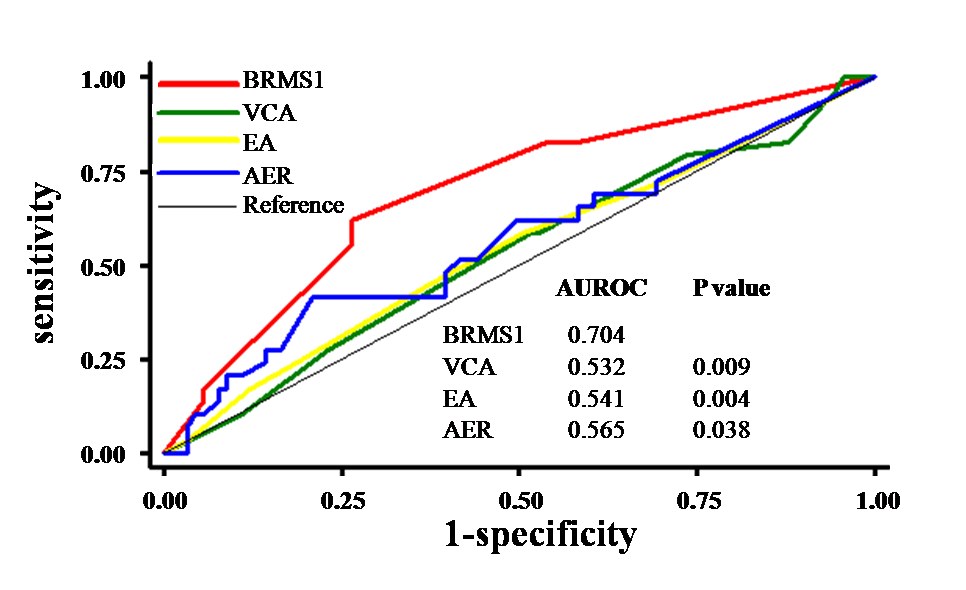

Supplement: Additional file 1 — Figure S1. [file 1471-2407-12-376-S1.tiff]
